# Tree age affects carbon sequestration potential via altering soil bacterial community composition and function

**DOI:** 10.3389/fmicb.2024.1379409

**Published:** 2024-07-09

**Authors:** Fengfeng Ma, Yang Liu, Youxiang Qi, Nan Deng, Huahao Xiang, Chuanlei Qi, Pai Peng, Liming Jia, Xuan Zhang

**Affiliations:** ^1^State Key Laboratory of Efficient Production of Forest Resources, Beijing, China; ^2^Key Laboratory for Silviculture and Conservation of Ministry of Education, Beijing Forestry University, Beijing, China; ^3^State Key Laboratory of Utilization of Woody Oil Resource, Hunan Academy of Forestry, Changsha, Hunan, China; ^4^Hubei Key Laboratory of Petroleum Geochemistry and Environment, Yangtze University, Jingzhou, Hubei, China; ^5^Zhilan Eco-environment Construction Limited Company, Changsha, Hunan, China

**Keywords:** *Larix kaempferi*, forest management, rhizosphere microbiome, soil organic carbon, tree stand age

## Abstract

Among various factors related to the forest carbon pool, the tree stand age, which interacts with soil organic matter, decomposition rates, and microbial activity, is essential and cannot be disregarded. However, knowledge about how tree phases influence soil carbon sinks is not adequate. This study sampled *Larix kaempferi* (Japanese larch) plantations with different tree stand ages to investigate the temporal dynamics of soil carbon sink in the forest. Physiochemical analyses and high-throughput sequencing results further revealed the interactions of tree stands and their related rhizosphere microbiome. It was found that microbial composition and metabolic activity were significantly affected by different tree ages, whose structures gradually diversified and became more stable from young to mature forests. Many keystone taxa from the phyla Chloroflexi, Proteobacteria, Acidobacteriota, and Nitrospirota were found to be associated with carbon transformation processes. Interestingly, the carbon resource utilization strategies of microbial groups related to tree ages also differed, with near-mature forest soils showing better labile carbon degradation capacity, and mature forests possessing higher degradation potential of recalcitrant carbon. Age-altered tree growth and physiology were found to interact with its rhizosphere microbiome, which is the driving factor in the formation and stability of forest soil carbon. This study highlighted that the tree age-associated soil microbiomes, which provided insights into their effects on soil carbon transformation, were significant in enhancing the knowledge of carbon sequestration in *L. kaempferi* plantations.

## 1 Introduction

Soil organic carbon (SOC) plays a crucial role in mitigating global climate change, the stability of which can decide the formation of non-labile C pools that are relatively difficult to decompose and release over a long period (Jiang et al., 2014). There are 45% of the forest C stock estimated at 662 Gt stored in soil organic matter (SOC) (Mäkipää et al., 2023), accounting for a significant portion of the C sequestration in the terrestrial ecosystem and an essential component of the global C cycle (Pan et al., 2011). Studies have shown that forest management contributes to the procedure for SOC formation and stability (Don et al., 2011); therefore, developing forest management-oriented climate change mitigation approaches is necessary for controlling greenhouse gas (GHG) emissions (Sasaki, 2021).

Many forest-related factors are involved in mitigating climate change and GHG emissions. Plant-derived C is an important contributor to C accumulation in forest ecosystems (Peichl and Arain, 2006). Studies usually emphasize the effects of biomass, species diversity, stand spatial structure, and woody products on forest C stock (Hui et al., 2019). For instance, forest-based activities have the potential to fulfill a mitigation amount of 441 Mt. CO_2_/year by 2050 through “climate-smart forestry” in the EU (Nabuurs et al., 2017). Researchers realized that carbon reallocation in the ecosystem was influenced by forest spatial structure, and the increase in carbon stocks was owing to tree species diversity enhancement (Forrester et al., 2021). Meanwhile, studies have also shown that soil C sequestration was evaluated at 9% of the overall mitigation potential of the forest ecosystem (Bossio et al., 2020). However, studies on forest soil in mitigating climate change were not sufficient, except for those studies focusing on afforestation, forest restoration, and avoidance of deforestation (Eriksson, 2020). More knowledge is needed on the mechanism of forests affecting SOC formation and stability.

Microbes are significant connectors between plants and soil (de Vries and Wallenstein, 2017). In particular, in the rhizosphere, plant residues and root exudates enabled microbial communities to have easy access to plant-derived carbon (Lange et al., 2024), which provided a rich available energy source for their resident microbiome to grow. In this sense, rhizospheric microbes are the most active microbial community for C sequestration in forest soil as they are actively involved in organic matter dynamics, nutrient cycling, and decomposition processes (Song et al., 2021). However, plant-derived carbon accessible for microbial community undergoes complicated mechanisms as plants usually experience spatial and temporal dynamics (Vetterlein et al., 2020), which lead to different compositions, quantities, and availabilities of rhizodeposits and root exudates. Therefore, analyzing the plant–microbe interactions could contribute to a deeper insight into how forest-derived carbon affects soil C stock.

Soil microorganisms played a crucial role in labile soil C pools (Patoine et al., 2022). They were regarded as decomposers of SOC in the previous research. Microbes directly influence the stability and release rate of organic C from labile C pools by decomposing OM and participating in processes such as carbon mineralization (Dai et al., 2017). However, recent studies have emphasized that soil microorganisms can not only induce organic C loss but also assist SOC formation and persistence (Schimel and Schaeffer, 2012). Soil microorganisms convert readily decomposable soil substrates into microbial biomass and metabolites through anabolism by “*in vivo* turnover” (Zheng et al., 2021). After the death of soil microorganisms, their necrotic material and some metabolites will remain relatively stable in the soil and contribute to the soil C pool in the form of microbial residues (Liang et al., 2017).

During the above carbon transformation process, forest management methods are important influencers to change the stability of SOC via altering microbes (Koné and Yao, 2021). It was observed that forest management can change microbial biomass, soil respiration, and microbial metabolic function diversity (Zheng et al., 2021). For example, increments of shrubs were found to increase soil respiration and microbial biomass carbon, as calculated by the qCO_2_ index, a sensitive indicator that was positively correlated to microbial biomass (Drum et al., 2019). Other research studies also found that forest management of *P. tabulaeformis* plantation altered microbial biomass, which became the major factor driving the stability of SOC (Song et al., 2021). Although it was well recognized that forest management practices affected microbial characteristics, the uncertainties of those effects still lay in the shifts of plant carbon allocation.

In addition, the tree stand age, which interacts with soil organic matter, decomposition rates, microbial activity, etc., cannot be disregarded as a significant impact factor on soil C pool accumulation (Li et al., 2023). For instance, young forests usually produce more biomass during the growing season. Mature forests had obvious rhizodeposition after the cold season. An investigation on four Masson pine plantations at 12-year intervals (Justine et al., 2017) found that the SOC content increased with age, and tree age became a controlling factor of photosynthetic and biological processes, thus affecting biomass accumulation and C sequestration in the plantation. Moreover, the age of forest stand affected the succession and regeneration of plant communities and soil environments, which may lead to changes in the diversity and structure of soil microbial communities (Wu et al., 2020). In Eucalyptus plantations, obvious differences in the composition and structure of soil bacteria and fungi in different stand ages were found (Qu et al., 2020). In Chinese fir plantation, stand age showed limited effects on bacterial composition but significantly increased the community diversity at the 17-year-old stand (Liao et al., 2022). However, the response mechanisms of soil C pools at different stand ages are complex and not well quantified. Moreover, information about plant–microbe interactions influenced by different forest stands is very limited. Therefore, unveiling the plant physiological changes and their correlations with microbial community characteristics are critical for effective forest management and the preservation and expansion of soil C stocks.

In this study, the plant–microbe interactions affected by forest ages were comprehensively investigated. Through physiochemical detection and 16S rDNA high-throughput sequencing, the structure, composition, and metabolic function of rhizosphere microbial communities of larch trees with different stand ages were analyzed. Combined with the soil and plant properties, the mechanism affecting SOC stability and plant-derived C allocation was further revealed. We hypothesized that (1) microbial community structure and SOC stability would differ under varied forest ages and (2) changes in microbial characteristics (including microbial biomass, microbial community structure, and microbial C metabolic pathways) would affect soil recalcitrant C production and SOC stability. The results are expected to provide information on plant–microbe interactions, which influence the forest soil C sequestration and mitigation of climate change.

## 2 Materials and methods

### 2.1 Site description

**Figure 1:**
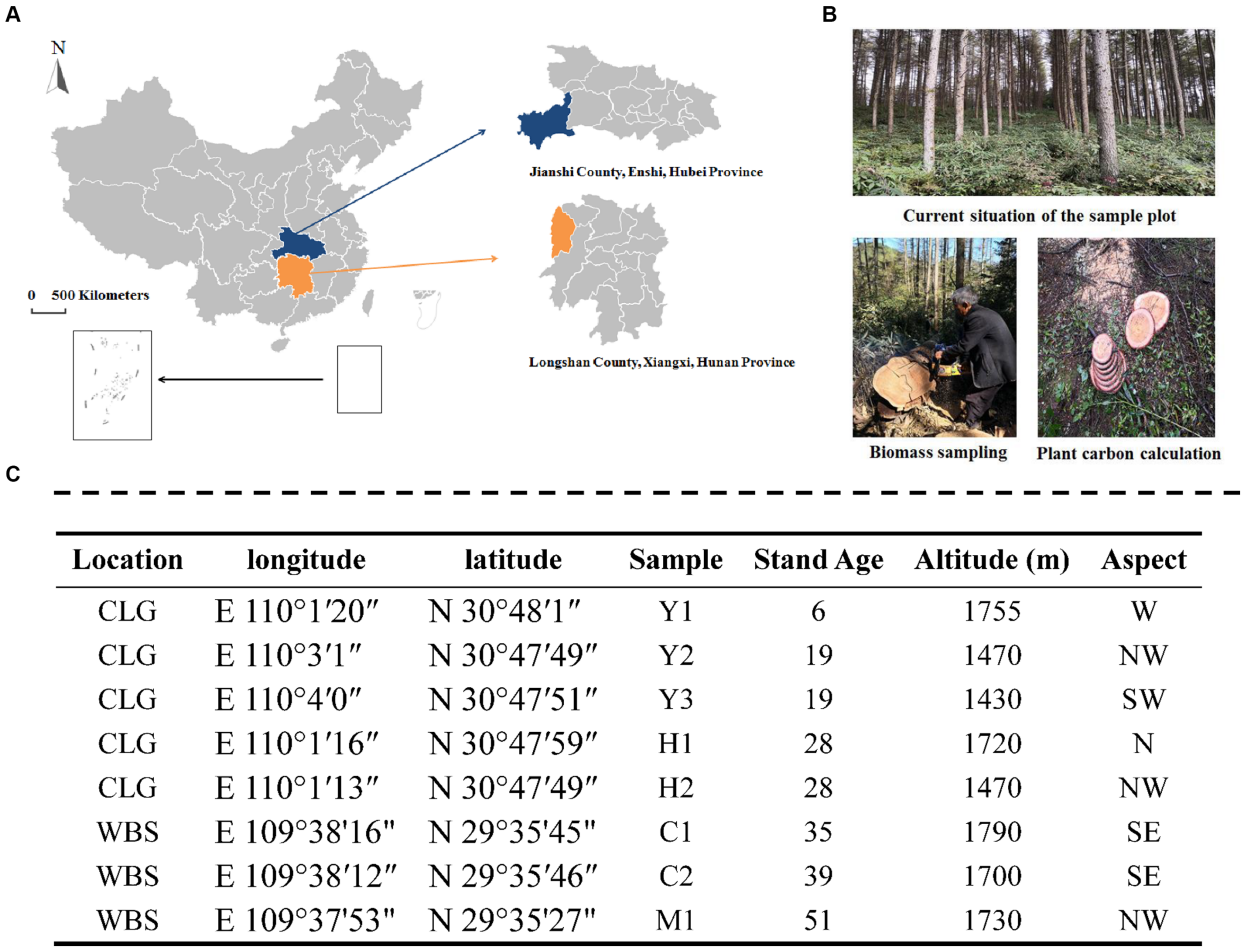
Location of the study site **(A)**, the image of plots **(B)**, and sampling design **(C)**. CLG forest farm (Jianshi County, Enshi, Hubei Province, China); WBS forest farm (Longshan County, Xiangxi, Hunan Province, China). Young stand (Y1, Y2, and Y3); middle-aged stand (H1 and H2); near-mature stand (C1 and C2); mature stand (M1); N, north; S, south; W, west; E, east.

*Larix kaempferi* (Japanese larch) is a species of larch native to Japan and widely used for forestry plantations across central and northern China, Japan, and Europe. It is a medium-sized to large deciduous coniferous tree, known for the tough and durable wood used mainly for construction work. The sampling sites were located in Changlinggang Forest Farm (30°48’ N, 110°0′ E) and Wanbaoshan Forest Farm (29°35’ N 109°38′ E), which represented typical central south areas of China (Figure 1). The two farms had similar climates, topography, altitude, and stand density. The forest stands are located in the same area, and the average annual temperatures of the forest stands are 11.2–11.7°C. The detailed characteristics of these stands are shown in Figure 1.

### 2.2 Experimental design and sample collection

Four stand types, including young- (6 and 19 years old), middle- (28 years old), near-mature- (35 and 39 years old), and mature-aged (51 years old) stands from eight locations (Figure 1) with close-to-nature management, were selected for the experiment. Three plots for each location were set as replications. The larch plantations chosen were all 2-year-old live seedlings at the time of planting. Sample collection took place in July 2018 to minimize heterogeneity due to climatic conditions and to ensure that plants reached their maximum growth rate during this period, which allowed us to assess the active and abundant bacterial community in the rhizosphere. Soil cores with an internal diameter of 38 mm were collected randomly in each plot at a depth of 20 cm using a multipoint method (*n* > 5). The soil samples were thoroughly mixed and transported in self-sealing bags to the laboratory for preservation. After removing rocks, roots, and other debris, the collected soil samples were sieved through a 2 mm sieve and divided into three parts. One part was air-dried and sieved through a 0.25-mm sieve to analyze the basic physical and chemical properties of the soil. The second part was stored at 4°C for the determination of soil microbial biomass and soil enzyme activities within 2 weeks. The third part was stored at −80°C for 16S gene sequencing.

Tree samples from each plot (400–600 m^2^) were measured for breast height (DBH) and height (H). The DBH and H of each standing tree were measured by calipers, diameter tapes, and height indicators. Total biomass (W) was calculated by the standard wood method, layered cutting, and the full root excavation method. Carbon storage per plant (Cplant) was measured according to Vinh et al. (2019). In detail, stem, branch, and leaf parts were collected, and the disks used for biomass calculation were intercepted to determine the Cplant based on the carbon content (CC_j_) according to Ma et al. (2016) and the biomass allocated from each organ and carbon layer (*B*_j_). The formula is as follows:


$$\mathrm{AGBCC}=\sum B_{j}*CC_{j}\text{,}$$

where AGBCC refers to the carbon content of the aboveground biomass (t), *B*_j_ is the biomass allocation to component, and CC_j_ is the carbon content to component (%).

### 2.3 Soil analysis

Soil samples were air-dried at room temperature, ground to powder, and sieved through 80 mesh (<0.18 mm) for further chemical measures. We measured the levels of soil organic carbon (SOC) and organic matter (OM) through dichromate oxidation and titration using ferrous sulfate (Mustapha et al., 2023). We used the drying method to measure the natural moisture content of soil and the ring knife method for measuring soil bulk density (BD), total porosity, capillary porosity, non-capillary porosity, and related water-holding characteristics (Shi et al., 2022). We determined microbial biomass carbon (MBC) content using chloroform fumigation and the K_2_SO_4_ extraction methods. The determination of soil catalase (CAT) and sucrase (SUC) was carried out using the potassium permanganate titration method and the colorimetric method (Fan et al., 2023), respectively. The determination of total nitrogen (TN) is carried out using the Kjeldahl method (Fan et al., 2023).

### 2.4 Statistical analysis

Amplicon sequence variables (ASV) are the same markers artificially assigned to a taxonomic unit (strain, genus, species, grouping, etc.) in phylogenetic or population genetics research for the purpose of analysis (Jia et al., 2021). Through platforms (Qiime v1.8.0) and software (vsearch 2.7.1), bioinformatics analysis was conducted on ASVs at a 97% similarity level to calculate the microbial community structure and composition at the phylum, genus, and ASV levels (Edgar, 2013). Alpha diversity calculations include Chao, Shannon, goods coverage, and observed species, were conducted by Mothur (v.1.30.1) based on OTU ≥ 97% sequence similarity of 0.97 for analyses (Zhang et al., 2021). Principal component analysis (PCA), redundancy analysis (RDA), and variance partitioning analysis (VPA) based on Bray–Curtis dissimilarity were carried out using the R vegan package. The variance inflation factor (VIF) values were calculated to remove redundancy factors before RDA (variables with VIF > 12) (Huhe et al., 2017). Using the Spearman test method, the top 20 genus-level results with an absolute abundance of all samples were selected for correlation analysis, and the corresponding gates were used as the legend. The cutoff values for interspecies interactions are Spearman’s correlation > |0.6| and *p* < 0.05 for filtering underperforming ASVs and reducing the complexity of the network (Barberán et al., 2012). Phylogenetic Investigation of Communities by Reconstruction of Unobserved States v2 (PICRUSt), a software to predict functional abundance based on marker gene sequences, was used to predict functional genes of 16S high-throughput sequencing data (Yao et al., 2022). Molecular ecological networks were constructed based on the relative abundance of ASVs in different treatments (Zhou et al., 2010). All network analyses (based on ASVs) were performed through the Molecular Ecological Network Analysis Pipeline (MENAP)^1^. Gephi 0.9.1-beta (Fan et al., 2024) was used for visualizing network interactions. Intra-module connectivity (Zi) and inter-module connectivity (Pi) were calculated in the coexistence network (Fan et al., 2024). Species were classified into four categories: (i) peripheral nodes (zi ≤ 2.5, Pi ≤0.62), which have very few links and are almost always connected to nodes within their own module, (ii) connectors (zi ≤ 2.5, Pi >0.62), which are highly connected to multiple modules, (iii) module hubs (zi > 2.5, Pi ≤0.62), which are highly connected to many nodes within their many nodes within the connected modules, and (iv) a network hub (zi > 2.5, Pi>0.62), which acts as a module hub and connector. As network hubs, module hubs, and connectors play an important role in maintaining the structure and functionality of the network, they are considered to be keystone taxa in the ecological network.

## 3 Results

### 3.1 Changes in soil physicochemical properties

#### 3.1.1 Plant growth parameters and carbon storage levels

This study calculated the changes in tree biomass and carbon storage of different tree ages (Table 1). Generally, the total biomass and individual carbon storage increased as the tree stand grew, with the maximum Cplant value (M1) being ~900 times over the minimum value (Y1). In addition, in terms of the carbon stock increase potential, the stage from middle-aged to mature forests has the highest rates of increase in W and Cplant, which is favorable to the accumulation of carbon pools.

**Table 1:** Plant growth level of *Larix kaempferi* (Japanese larch) plantation.

Sample group	Stand age (year)	Parse tree		*W* (kg)	Cplant (t)
		DBH (cm)	*H* (m)		
Y1	6	5.43 ± 0.45	8.00 ± 0.400	4.70 ± 1.05	0.0028 ± 0.00
Y2	19	12.03 ± 1.18	12.00 ± 0.69	38.75 ± 10.69	0.0231 ± 0.01
Y3		13.43 ± 1.40	13.13 ± 0.55	53.39 ± 12.50	0.0319 ± 0.01
H1	28	18.00 ± 1.04	23.17 ± 2.21	148.18 ± 29.88	0.09 ± 0.020
H2		15.27 ± 0.60	14.33 ± 0.75	75.71 ± 2.22	0.05 ± 0.00
C1	35	30.27 ± 2.35	25.07 ± 1.40	572.34 ± 135.34	0.34 ± 0.08
C2	39	28.07 ± 1.65	28.17 ± 0.70	543.98 ± 102.17	0.33 ± 0.06
M1	51	50.83 ± 1.31	35.10 ± 1.40	4211.92 ± 548.7	2.53 ± 0.33

Young stand (Y1, Y2, and Y3); middle-aged stand (H1 and H2); near-mature stand (C1 and C2); mature stand (M1); W (total biomass); Cplant (carbon storage per plant).

#### 3.1.2 Soil physicochemical properties of different forest stands

With increasing forest age, there was a growing tendency for the soil minimum water-holding capacity (Wmin) to peak in the mature group (M) at 74.42% (Table 2). SOC and OM showed a significant increasing trend with the growth of forest age, reaching a peak in near-mature forests at 46.70 and 80.51 g/kg, respectively, and then decreasing in mature forests. The soil enzymes were further detected to examine microbial metabolic activity. Similar to the trends of SOC and OM, the microbial biomass carbon (MBC) and catalase (CAT) concentration reached the highest levels in the near-mature stand group (C) (Table 3).

**Table 2:** Soil physicochemical parameters of different sampling groups.

Soil parameters	*Y*	*H*	*C*	*M*
Wmax (%)	72.32 ± 26.32	68.26 ± 30.16	74.58 ± 0.48	78.56 ± 11.35
Wmin (%)	62.41 ± 18.13	64.54 ± 26.79	68.1 ± 0.26	74.42 ± 9.33
BD (g/cm3)	0.86 ± 0.28	0.90 ± 0.24	0.83 ± 0.04	0.78 ± 0.14
SOC (g/kg)	30.11 ± 10.68	21.9 ± 5.13	46.7 ± 12.45	32.22 ± 15.38
OM (g/kg)	51.9 ± 18.42	37.75 ± 8.85	80.51 ± 21.45	55.54 ± 26.52
MBC (mg/kg)	0.26 ± 0.01	0.22 ± 0.09	0.37 ± 0.14	0.25 ± 0.08
CAT (μmol/d/g)	37.83 ± 1.69	36.00 ± 1.85	42.25 ± 1.85	31.74 ± 1.11
SUC (μmol/d/g)	25.23 ± 22.20	15.75 ± 11.70	10.55 ± 0.49	8.79 ± 7.89

Young stand (Y); middle-aged stand (H); near-mature stand (C); mature stand (M); Wmax (maximum water-holding capacity); Wmin (Minimum water-holding capacity); BD (bulk density); SOC (soil organic carbon); OM (organic matter); MBC (microbial biomass carbon); CAT (catalase); SUC (sucrase).

**Table 3:** Topological properties of the empirical MENs of microbial communities in the Y, H, and C groups.

Communities	*Y*	*H*	*C*
No. of original ASVs	100	100	100
Similarity threshold (st)	0.6	0.6	0.6
Total nodes	100	100	100
Total links	1,658	679	483
Avg. path length (GD)	1.892	2.895	3.089
Avg. clustering coefficient (avgCC)	0.713	0.593	0.5
No. of modules	3	5	4
Modularity	0.413	0.513	0.493

Young stand (Y); middle-aged stand (H); near-mature stand (C).

#### 3.1.3 Correlations between SOC and environmental properties

**Figure 2:**
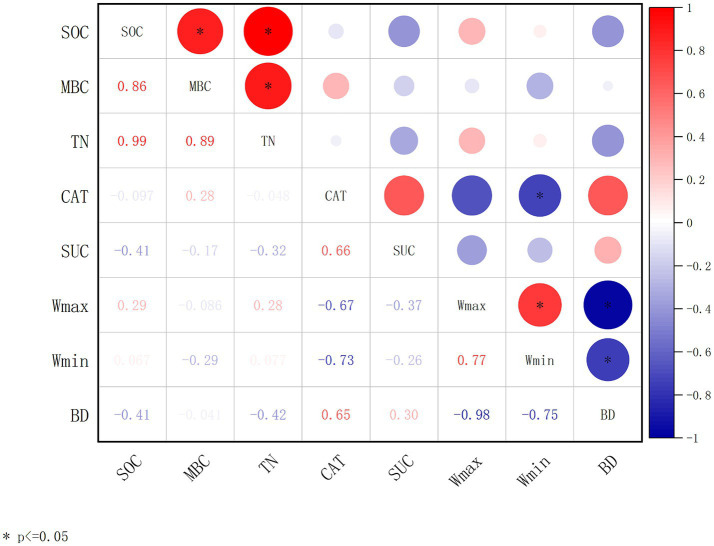
Correlation analysis of soil parameters and microbial enzyme activities. The correlation coefficient is obtained by calculating Pearson’s correlation coefficient, which is used to reflect the interrelationship between the variables. Soil organic carbon (SOC); microbial biomass carbon (MBC); total nitrogen (TN); catalase (CAT); sucrase (SUC); maximum water-holding capacity (Wmax); minimum water-holding capacity (Wmin); bulk density (BD). The correlation coefficients are indicated by hue. *p < 0.05, **p < 0.01, ***p < 0.001.

Changes in environmental factors often cause significant disturbances to the soil carbon pool. The relationships among SOC and soil and microbial parameters are complex. Plant growth and mortality processes and microbial activities in the soil are the main factors affecting SOC accumulation. As shown in Figure 2, from the correlation analyses of SOC with soil physiochemical factors (TN, Wmax, Wmin, and BD) and microbial metabolic activities (MBC, CAT, and SUC), it was obvious that SOC has significant positive correlations with microbial carbon (MBC) and total nitrogen (TN). TN was also significantly positively correlated to MBC (Figure 2).

### 3.2 Alpha and beta diversities of different bacterial communities

**Figure 3:**
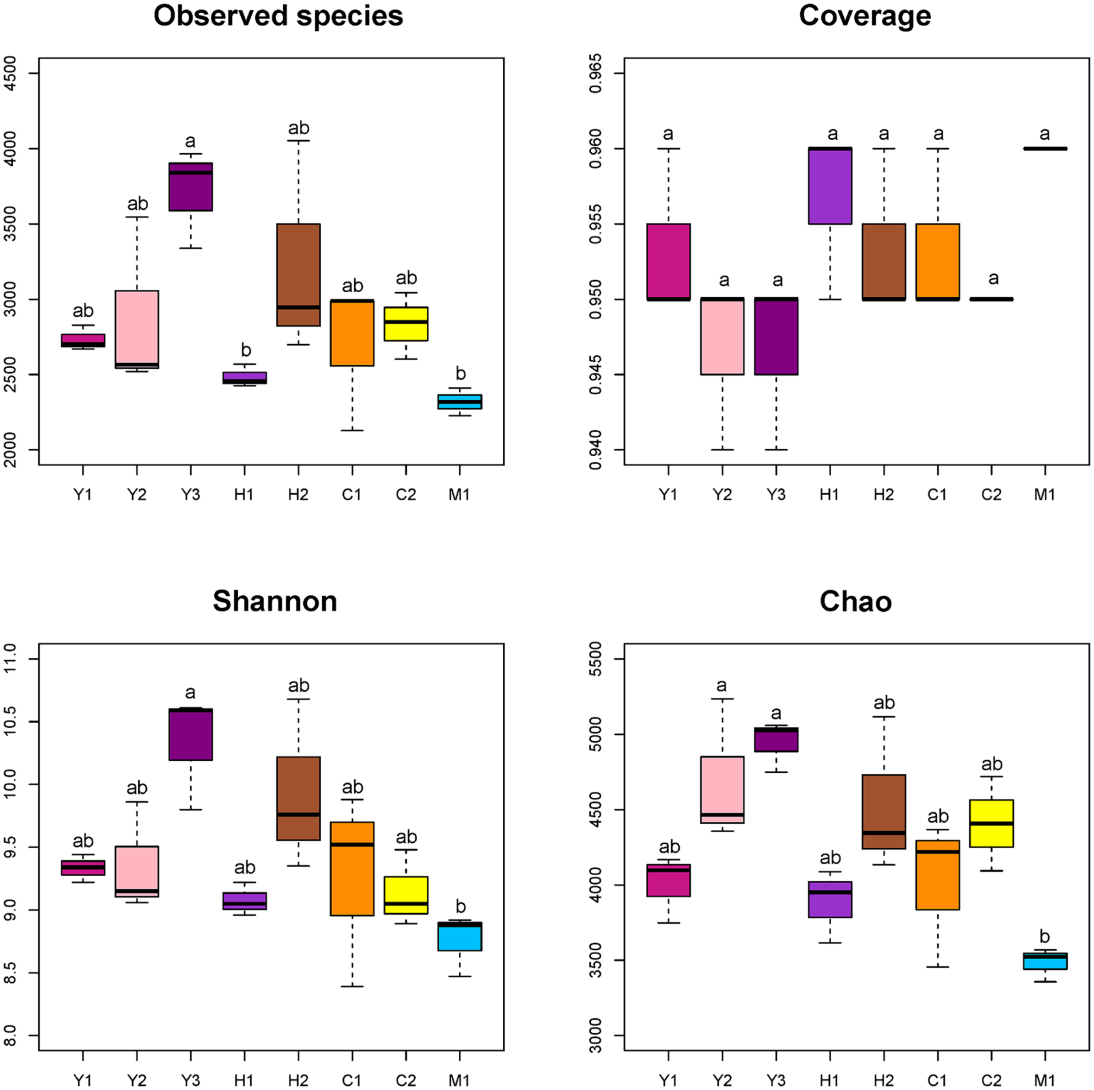
Alpha diversity indexes of different bacterial communities. Observed species: no. of species actually observed. Coverage: degree of species coverage. Shannon: reflects community diversity index. Chao: species abundance. Young stand (Y1, Y2, and Y3); middle-aged stand (H1 and H2); near-mature stand (C1 and C2); mature stand (M1).

Quality control of sequences was conducted by filtering, merging, and removing host plant gene sequences. A total of 2,797,181 clean reads were obtained with a median distribution at 200–260 bp lengths. Sequences were further clustered into 9,216 ASVs, ranging from 2,129 to 4,053 ASVs in individual samples with rarefaction curves of each sampled sequence (Supplementary Figure S1). Alpha diversity was evaluated on an ASV level through observed species, coverage, diversity, and richness. As shown in Figure 3, the species obtained demonstrated an increasing trend followed by a declining trend as tree age grew, which showed the highest number in the young stand group (Y2) and the lowest in the mature stand group (M1). The coverage of each sample ranging from 0.94 to 0.96 was close to 1 representing good sequencing accuracy (Figure 3). In accordance with the species number, Shannon and Chao indexes, which represented the diversity and richness of microbial community samples (Figure 3), showed increasing then decreasing tendency with the tree age getting older.

**Figure 4:**
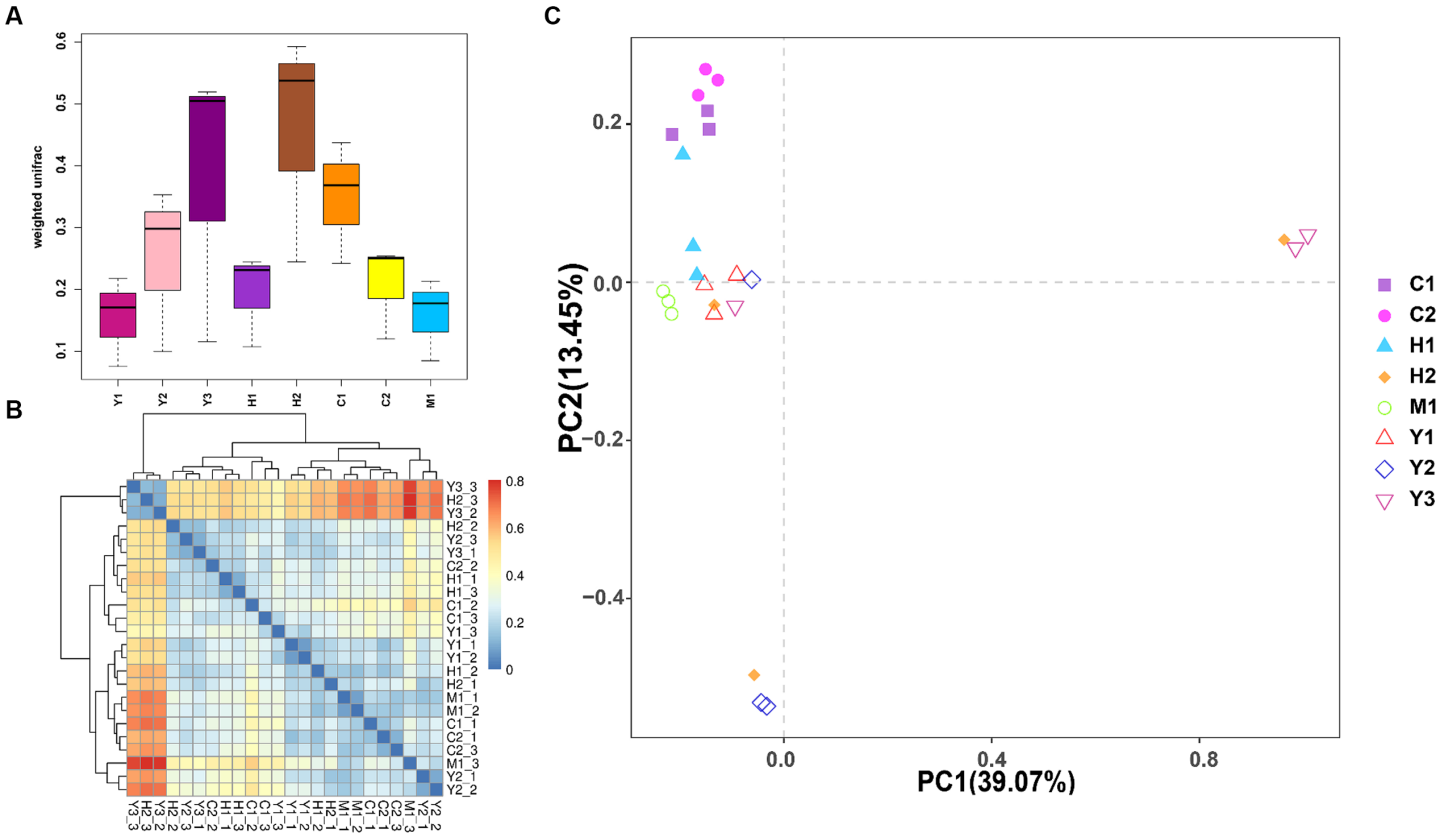
Weighted UniFrac distance between different samples shown by boxplot **(A)** and heatmap **(B)**. The samples are color-coded according to the similarities between each of them: from zero (blue) to over (red). Principal component analysis (PCA) **(C)**: the values of PC1 and PC2 are the percentages that can be explained by the relevant axis.

Beta diversity of the bacterial community was further revealed through UniFrac distance (Figures 4A,B). It was found that the distances among bacterial communities of young forests (Y1, Y2, and Y3) were longer than those of near-mature (C1 and C2) and mature (M1) forests, indicating more variations within the group of young stands than older stands. Except for UniFrac distance calculation, the PCA (Figure 4C) could better reveal similarities and dissimilarities among the structure of microbial communities through the sample spot distribution in the corresponding axes. Moreover, tree age shaped the structure of the bacterial community to some extent as the sample spots scattered according to their age groups. Near-mature groups C1 and C2 clustered together, and a sample of the mature group (M1) gathered tightly. However, the groups of young age Y1, Y2, and Y3 were distributed separately, indicating that the structure of young forest microorganisms varied more fiercely than those of close-to-mature age.

### 3.3 Bacterial community composition and dominant species of different samples

**Figure 5:**
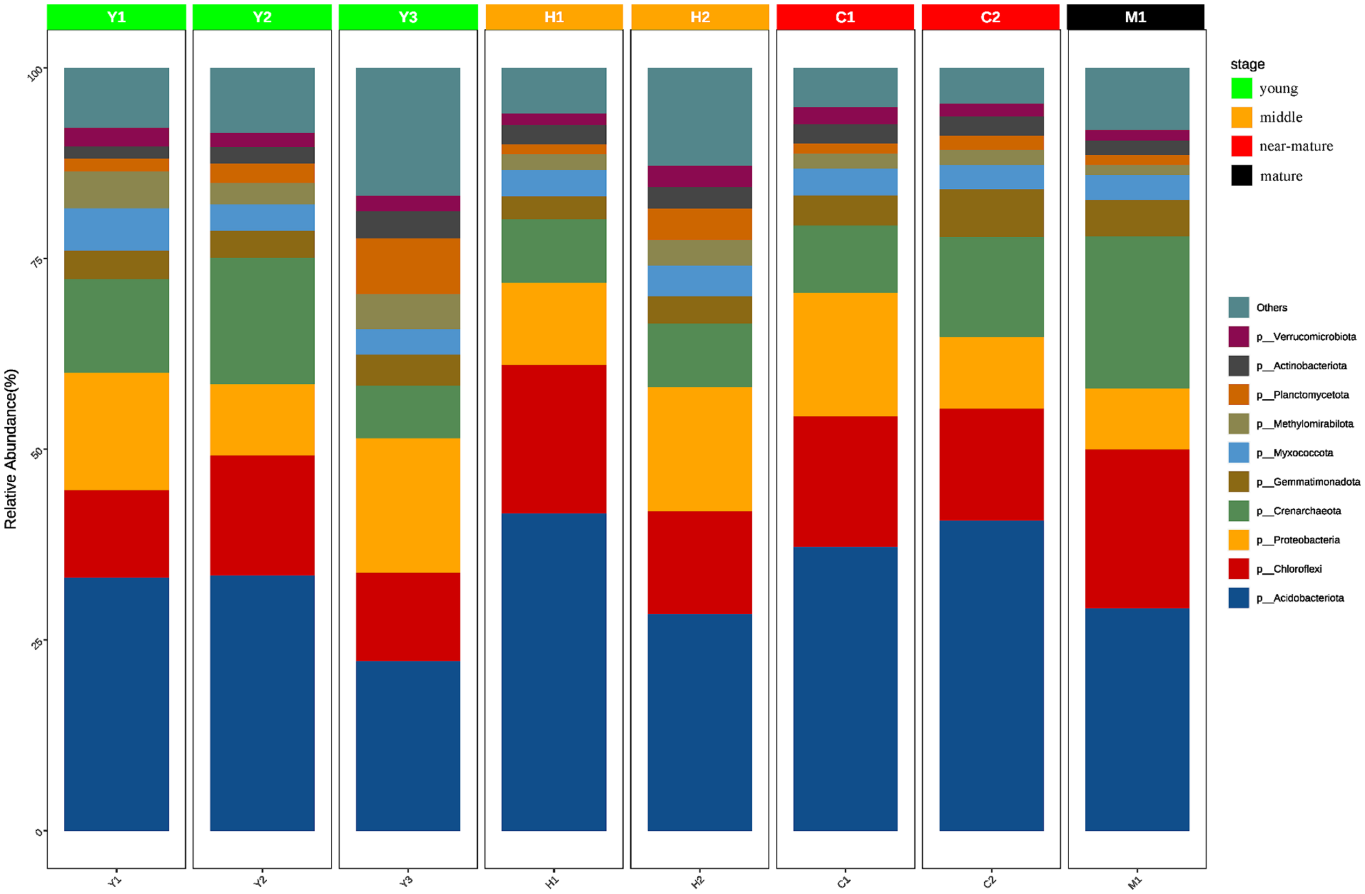
Species composition of different groups at the phylum level. The horizontal axis represents the sample name, and the vertical axis represents the relative abundance of the species in the sample. The figure shows species information with relative abundance of over 1%.

To further understand the community structure and composition of microorganisms, statistical analysis of the ASVs obtained from the samples revealed a total of 43 phyla with relative abundance greater than 1%. Actinomycetes, a group of bacteria that are widely present in the soil and play an important role in the soil carbon cycle and have a significant impact on soil organic matter decomposition, carbon transformation, and degradation of organic matter, were the most common phylum in the samples with an average of 33.24% across all samples. Other dominant phyla included Chloroflexi (15.54%), Proteobacteria (12.87%), and Crenarchaeota (11.80%). Despite the dominance of these phyla in all samples, some differences in community structure were observed in the different fractions of samples (Figure 5).

It was discovered that the community abundance of dominant phyla changed significantly and the community abundance of non-dominant phyla increased significantly during the time series from young forests (Y1, Y2, and Y3) to middle-aged forests (H1 and H2). In terms of community abundance, middle-aged forests (H1 and H2) had a more diversified community structure compared with near-mature forests (C1 and C2), while near-mature forests (C1 and C2) and mature forests (M1) were similar in terms of community structure and abundance and gradually tended to be in a stable state. Changes in tree age shaped microbial community structure and abundance to a certain extent, and the microbial community structure of young (Y1, Y2, and Y3) and mature (M1) forests changed from diversified to more stable.

### 3.4 The relationship between soil properties, plant growth levels, and microbial metabolic activities

**Figure 6:**
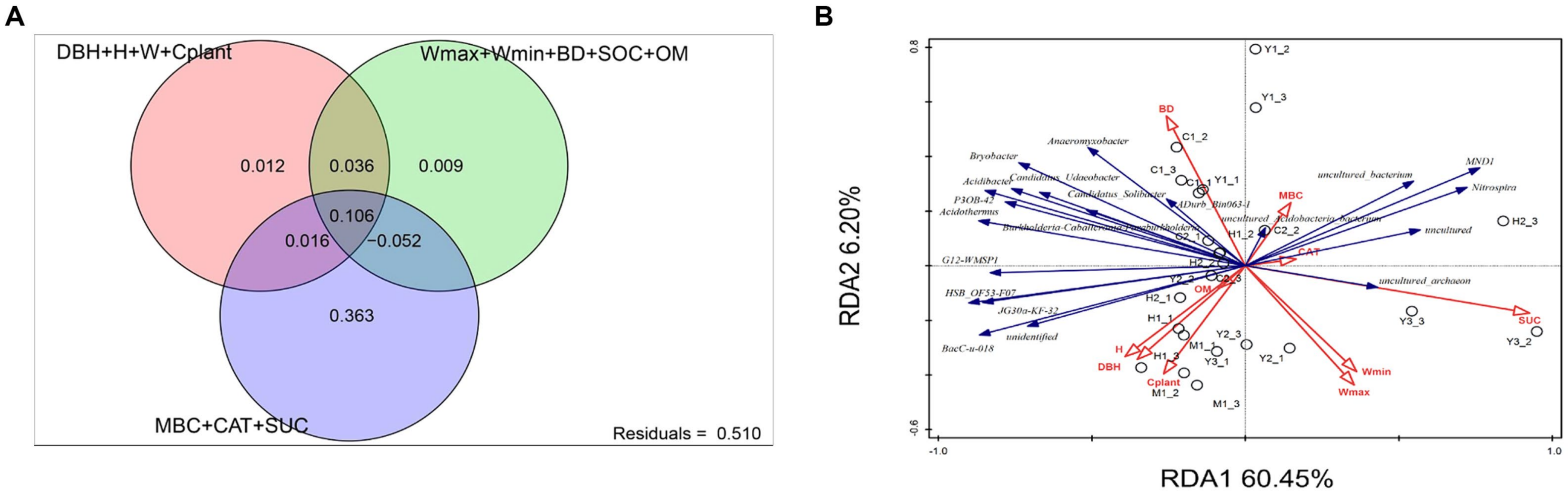
Variance partitioning analysis (VPA, **A**) and redundancy analysis (RDA, **B**). Variables presented in the RDA plot were selected by variance inflation factors. For VPA, variables presented in RDA were separated into three groups: plant growth level (DBH, H, W, and Cplant), soil physicochemical factors (Wmax, Wmin, BD, SOC, and OM), and microbial metabolic level (MBC, CAT, and SUC).

The investigation of environmental factors could help us to further explain variations among bacterial communities affected by the environment. After eliminating correlated variables based on variance inflation factors (VIF > 12), there were altogether 12 environmental factors chosen for RDA. The variance partitioning analysis (VPA) was conducted to classify and calculate the importance of those 12 factors toward microbiome structures. The 12 variables were classified into three categories: microbial metabolic activities (MBC, CAT, and SUE), soil properties (Wmax, Wmin, BD, SOC, and OM), and plant growth parameters (DBH, H, W, and Cplant). Figure 6A shows microbial metabolic activities that explained 36.3% of the bacterial community structure independently, which was the primary influencer compared to plant and soil factors. RDA, as presented in Figure 6B indicated that mature forest (M1) was significantly correlated with the plant growth factors, especially plant carbon stocks (Cplant). The microbial structure of close-to-mature forest (C1-3) had a significant correlation with soil parameters such as BD and microbial activity such as MBC.

### 3.5 Molecular ecology networks (MENs) and keystone taxa

#### 3.5.1 Topological properties of different MENs

**Figure 7:**
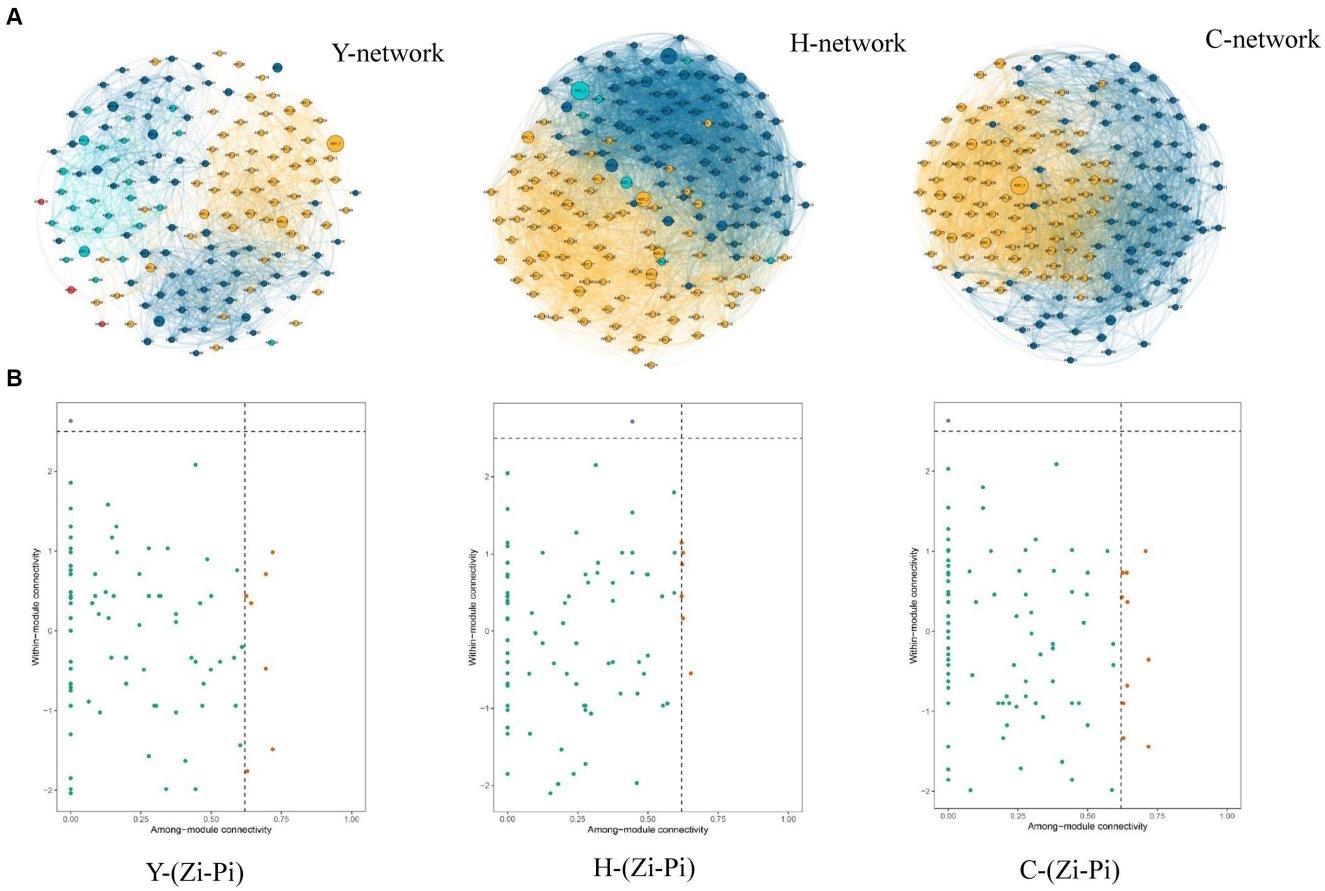
Co-occurrence network of microbial communities in each sample and node classification **(A)**. The size of the dots represents the magnitude of abundance, and the thickness of the line represents the magnitude of correlation; the color of the dots represents the phylum to which they belong, with a red line indicating positive correlation and a blue line indicating negative correlation. The Zi-Pi diagram showed the distribution of ASVs according to their Zi and Pi values based on the topological structure **(B)**. Network hubs: Zi >2.5, Pi >0.62; module hubs: Zi > 2.5, Pi ≤0.62; connectors: Zi ≤ 2.5, Pi >0. 62; peripheral nodes: Zi ≤ 2.5, Pi ≤0.62.

Molecular ecological network analysis can be used to reveal the interactions among species and the structural stability of each group (Jia et al., 2021). Three MENs based on the ASVs of each sample group were constructed to show the interspecies correlations of the microbial community of young stand (Y), middle-aged stand (H), and close-to-mature stand (C) (Figure 7A), and the corresponding topological properties are shown in Table 4. The three networks filtered data with *p*-values greater than 0.05 or correlation values of |R| > 0.6, using the corresponding gate as the legend, indicating that the three networks have typical hierarchical, small-world, and modular characteristics, which can be used for subsequent molecular ecological network analysis (Li et al., 2022). Compared to Y and C, MEN of H referring to middle-age forest had better modularity and more modules, while the MEN of Y, representing the young stand group, possessed more interspecies connections with 1.4- to 2.4-fold links than H and C.

**Table 4:** Affiliation of keystone taxa in the *Zi-Pi* graph.

Group		Nodes	Affiliations	Phylum
Y	Module hubs	ASV_66	C:AD3	Chloroflexi
	Connectors	ASV_25	G:Acidibacter	Proteobacteria
		ASV_18	C:Acidobacteriae	Acidobacteriota
		ASV_88	C:Acidobacteriae	
		ASV_26	G:Nitrospira	Nitrospirota
		ASV_103	C:Bathyarchaeia	Crenarchaeota
		ASV_57	F:Methanomethyliaceae	
		ASV_107	C:AD3	Chloroflexi
H	Module hubs	ASV_66	C:AD3	Chloroflexi
	Connectors	ASV_1	C:Nitrososphaeria	Crenarchaeota
		ASV_23	C:Bathyarchaeia	
		ASV_103	C:Bathyarchaeia	
		ASV_7	C:Acidobacteriae	Acidobacteriota
		ASV_68	C:Acidobacteriae	
		ASV_119	F:Ktedonobacteraceae	Chloroflexi
C	Module hubs	ASV_66	C:AD3	Chloroflexi
	Connectors	ASV_1	C:Nitrososphaeria	Crenarchaeota
		ASV_2	C:AD3	Chloroflexi
		ASV_48	C:AD3	
		ASV_55	C:TK10	
		ASV_46	C:AD3	
		ASV_6	C:Acidobacteriae	Acidobacteriota
		ASV_21	C:Acidobacteriae	
		ASV_25	C:Gammaproteobacteria	Proteobacteria
		ASV_51	G:Anaeromyxobacter	Myxococcota
		ASV_74	O:Rokubacteriales	Methylomirabilota

P, phylum; C, class; F, family; and G, genus.

#### 3.5.2 Keystone taxa identification

Different species, which are identified as nodes in the MENs, played respective ecological and functional roles (Gu et al., 2023). Identifying the keystone taxa in the network based on node Zi and Pi values is useful for exploring the niche and function of bacteria in various environments (Banerjee et al., 2018). The ASVs in the networks were classified into four categories based on their within-module connectivity (Zi) and among-module connectivity (Pi) values as shown in Figure 7B. The majority of the ASVs in each network were classified into peripherals. There were 1 module hubs in Y, H, and C networks, respectively. The nodes of connectors in the Y, H, and C networks were 7, 6, and 10, respectively. None of the nodes were classified into network hubs in any MENs. The phylogenetic classification of the keystone taxa (Table 4) showed that some members of the phyla Chloroflexi, Acidobacteriota, and Crenarchaeota were the prominent keystone taxa in all networks. The members of Chloroflexi, Proteobacteria, Acidobacteriota, Nitrospirota, Crenarchaeota, Myxococcota, and Methylomirabilota comprised the core microbiome. Co-occurrence network analyses revealed an overlap of the keystone taxa in Y, H, and C. In particular, Chloroflexi occupies crucial positions in all three molecular network module hubs, suggesting that it occupies an important ecological niche.

#### 3.5.3 Functional gene prediction and distribution

**Figure 8:**
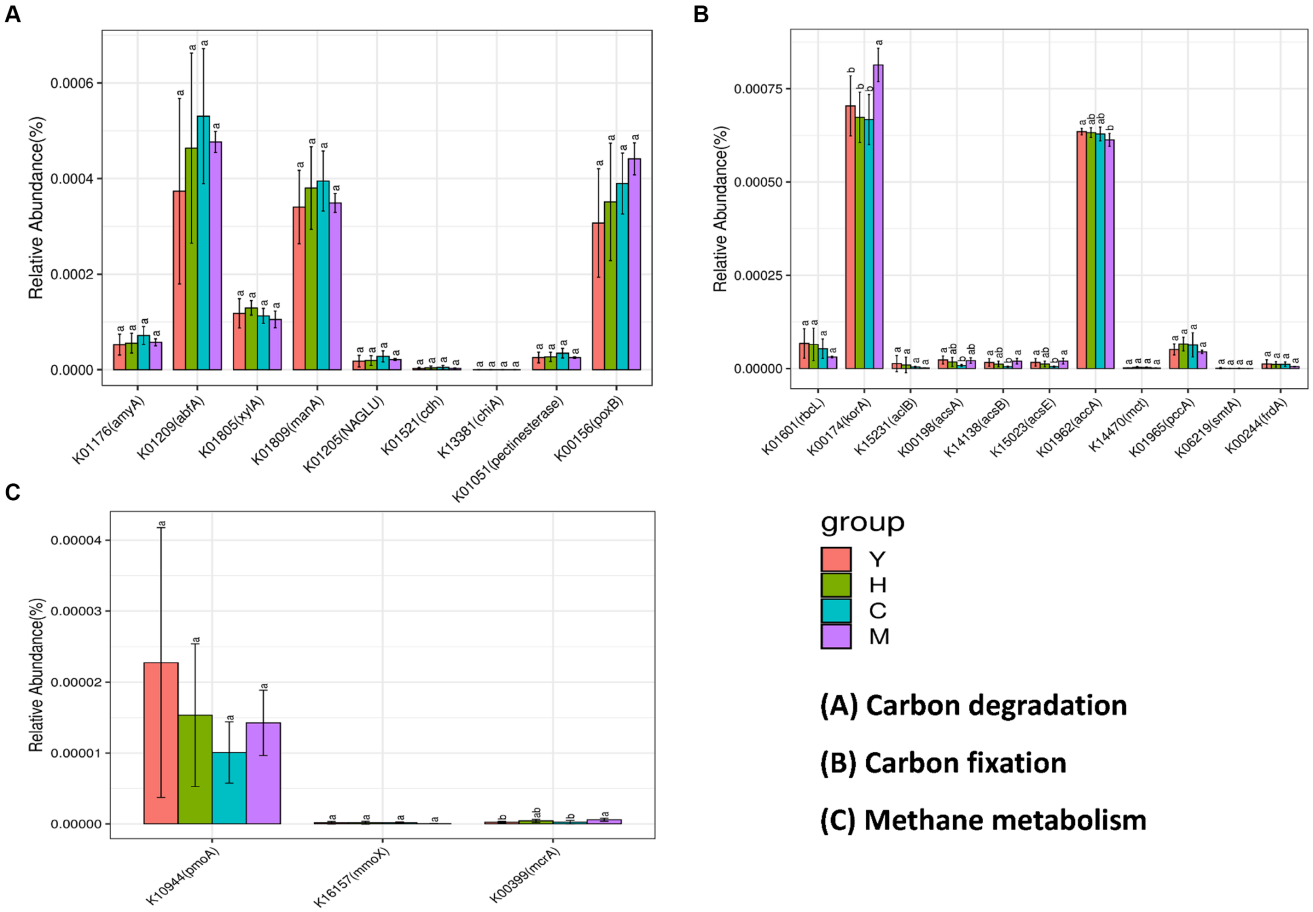
Abundance prediction of key functional genes in the carbon cycle process. Bacterial contributed genes related to carbon degradation **(A)**, carbon fixation **(B)**, and methane metabolism **(C)**. Different tree age groups of young stands (Y); middle-aged stands (H); Near-mature stands (C); and mature stands (M).

To further reveal the functional genes distributed in different sample groups and their interactions with ecological keystone taxa, PICRUst was utilized to predict the functional genes among different samples. KEGG orthologues (KO) identified in 16S rRNA amplicons and the number of genes per sample are shown in Figure 8. The soil carbon cycle processes in which microorganisms are involved are mainly carbon degradation (decomposition of organic matter), methane metabolism (methane production and methane oxidation), and carbon fixation (the conversion of carbon dioxide into organic matter). Among the carbon degradation-related functional genes (Figure 8A), abfA, manA, and poxB were the most abundant. The abundance of abfA and manA genes related to hemicellulose degradation showed a trend that increased with forest age, peaked in near-mature forest, and then declined in the mature forest stage. The abundance of pox, which is associated with lignin degradation, increased with age. The abundance of amyA, which is related to starch degradation and pectin degradation, both peaked at the near-mature forest stage. Methane metabolism is divided into two pathways, namely, methane generation and methane oxidation. The key functional genes of methane generation (pomA and mmoX), among which pomA possessed high gene abundance during methane metabolism, are shown in Figure 8C. The abundance of mcrA, a functional gene involved in methane oxidation, did not differ significantly between the four fractions. Figure 8B illustrates the abundance of functional genes responsible for soil microbial carbon fixation under varying time series conditions. The data show that korA and accA genes exhibit the highest abundance among all carbon fixation-related functional genes. The korA gene was significantly increased in mature forest stands, while accA was significantly increased in young forest stands. The rest of the genes related to carbon fixation did not show a significant difference.

## 4 Discussion

### 4.1 The effect of tree age on the formation of SOC

Age-related changes in tree growth and physiology are key factors affecting SOC accumulation (Groover, 2017), among which higher biomass generated during the lifespan of a tree could directly contribute to the accumulation of exogenous carbon inputs increasing SOC. The findings of this study on tree biomass and plant carbon stock were in accordance with previous studies that when trees become older, they generally get higher carbon stocks, as shown in Table 1 that Cplant followed the trend of young stand (Y1, Y2, and Y3) < middle-aged stand (H1 and H2) < near-mature stand (C1 and C2) < mature stand (M1) < mature stand (M1). To assess the effect of tree age on exogenous carbon inputs of SOC, we further explored SOC at each tree stage and its associated soil physicochemical properties and microbial metabolism activities. We found that SOC content did not maintain the same growing trend with tree age but rather peaked at the near-mature forest stage and then gradually decreased and stabilized at the mature forest stage (Table 2). This phenomenon might be attributed to the verified lifestyle during the growth and aging of larch. From juvenile to adult, trees often demonstrated differences in morphology and physiology. In detail, young stands may retain dead leaves after dormancy in the fall, while mature stands may prefer to let leaves be abscised and shed (Hallé et al., 2012). These characteristics brought by varied litters under the forest would affect the formation of SOC. From the scope of the whole plantation, the seasonal growth and community replacement also shaped the SOC of the forest system. For instance, a study investigated a ~70-year-old chronosequence of larch plantations and found that SOC stocks were significantly negatively correlated with litter and herb biomass, which suggested that thinning around 16 years should be conducted to provide enough space for litter decomposition and understory growth. In summary, trees in different growth stages usually require nutrients in different amounts, which would possibly lead to the corresponding decomposing rates of carbon sources driven by root system, thus influencing the processing of SOC formation and degredation (Wang et al., 2020).

### 4.2 Interactions between trees and their microbiome affected the stability of SOC

As rhizosphere microorganisms bridge trees and soil in carbon cycling, this study emphasized the interactive effects between temporal differential tree stands and the respective microbiome to reveal their mechanisms on SOC stability. The correlation analysis among SOC, soil nutrients, and microbial enzymes at different stand ages (Figure 2) demonstrated that SOC was strongly associated with microbial biomass carbon and total nitrogen. This phenomenon raises the possibility that microorganisms are active players in the soil carbon cycle and essential regulators of SOC under various tree age circumstances. It was inferred that as the age of the stand increases, the top soil of larch plantation forests produces more organic materials, altering the nutrient structure of the soil, leading to an increase in below-ground biomass and root exudates, which promotes soil microbial conversion and utilization, resulting in higher microbial biomass and metabolism levels (Table 2 and Figure 2). Apart from microbial biomass, this study also showed that tree age alters microbial community composition (Figures 5, 6) and function (Figure 8). From alpha and beta diversity analyses, it was shown that the bacterial community structure gradually diversified and became more stable from young to mature forests. In addition, along with the soil microbial biomass carbon increasing with the tree age growth (Table 2), catalase accordingly increased, and significant positive correlations were found among soil microbial biomass and sucrase and catalase (Figure 2), with sucrase being the most prominent one. All these phenomena suggested that variability in larch growth levels and stand environment under different age conditions led to differences in the amount of organic matter entering the soil, which, in turn, affected the nutrient content of microbial metabolic processes. Changes in nutrient content in the soil altered microbial abundance levels, structural composition, and metabolic functions, which ultimately affected the regulation of SOC.

### 4.3 Microbial functional gene and keystone taxa in larch rhizosphere to shape SOC

Functional genes are primarily involved in microbial biochemical processes, as well as mediating elemental cycling by encoding specific functional proteins, whose abundance can predict the potential of soil microorganisms for material cycling and metabolic characteristics. Interestingly, the results of this study showed different carbon resource utilization strategies of microbial groups related to tree ages. Specifically, functional genes related to hemicellulose degradation were found to possess the highest gene abundance during carbon degradation (Figure 8A). The strongest hemicellulose decomposition ability was found in near-mature forests and those of starch and chitin degradation genes. On the other hand, mature forest has a relatively strong potential to decompose difficult-to-decompose carbon, as shown by the genes related to lignin and cellulose degradation were more enriched in this group. Overall, near-mature forest soils had better performance on labile carbon degradation; meanwhile, mature forests possessed a higher potential for degradation of refractory carbon compared to other fractions. Furthermore, the carboxylic acid cycle is the final metabolic pathway of the three major nutrients (sugars, lipids, and amino acids) and the hub of the metabolic linkage between sugars, lipids, and amino acids, which plays an important role in microbial carbon fixation. The results of this study revealed that the mature forest group had the highest abundance levels of korA and accA related to carbon fixation (Figure 8B), suggesting that soil microorganisms in mature forests have a stronger ability to sequester carbon.

In addition to functional genes, keystone taxa dominated the core niche of the community and also played an important role in shaping the community structure and functions. Among the few keystone taxa, including Chloroflexi, Proteobacteria, Acidobacteriota, Nitrospirota, Crenarchaeota, Myxococcota, and Methylomirabilota, investigated through MEN’s analysis, Chloroflexi is not only a dominant phylum with an average of 15.54% of all forest age fractions (Figure 5) but also a keystone species occupying important ecological niches as revealed by molecular ecological network analysis (Table 4). Chloroflexi has a pathway for complete hydrolysis or oxidative degradation of a wide range of recalcitrant organic substances, and the 3-hydroxypropionate bicycle is an essential pathway for autotrophic and carbon fixation (Shih et al., 2017). PICRUst gene function prediction showed a high abundance of functional genes involved in carbon degradation of difficult-to-catabolize carbons, such as lignin and cellulose, which is consistent with the “feast or famine” metabolic strategy that Chloroflexi may follow (Liu et al., 2022). In addition, the abundance of functional genes during carbon fixation (Figure 8B) showed that korA, which is involved in the 3-hydroxypropionate bicycle, had the highest abundance, which was consistent with the CO_2_ fixation and autotrophic mode of Chloroflexi. It could be inferred that Chloroflexi occupies a vital ecological niche and is a crucial microflora in the carbon cycle process, with an outstanding degree of contribution to both carbon degradation and carbon fixation (Garritano et al., 2022).

## 5 Conclusion

Through a regional-scale sampling of the larch plantation across the central south of China, the spatial and temporal dynamics of the tree-related carbon sink and cycling were investigated. As the larch tree passed through juvenile to adult, vegetative and reproductive phases, the tree biomass significantly increased, allowing more exogenous carbon to enter the soil, which increased microbial biomass and metabolic activities, and finally led to significantly higher SOC accumulation at the near-mature and mature forest stages. The findings on microbial functional genes and keystone taxa can help with a deeper understanding of forest management toward better regulation of the forest C stock in the future.

## Data availability statement

The datasets presented in this study can be found in online repositories. The names of the repository/repositories and accession number(s) can be found in the article/Supplementary material.

## Author contributions

FM: Writing – original draft, Investigation, Formal analysis. YL: Writing – original draft, Formal analysis. YQ: Writing – original draft, Resources, Investigation. ND: Writing – review & editing, Software. HX: Writing – original draft, Visualization. CQ: Writing – review & editing, Investigation. PP: Writing – review & editing, Investigation. LJ: Supervision, Writing – review & editing, Conceptualization. XZ: Writing – review & editing, Conceptualization.

## Footnotes

^1^ http://129.15.40.240/MEAN/
