# Supplementary figures and images for: Tree age affects carbon sequestration potential via altering soil bacterial community composition and function
Source: Front Microbiol. 2024 Jul 9;15:1379409. doi: 10.3389/fmicb.2024.1379409 (PMC11265291; doi:10.3389/fmicb.2024.1379409)

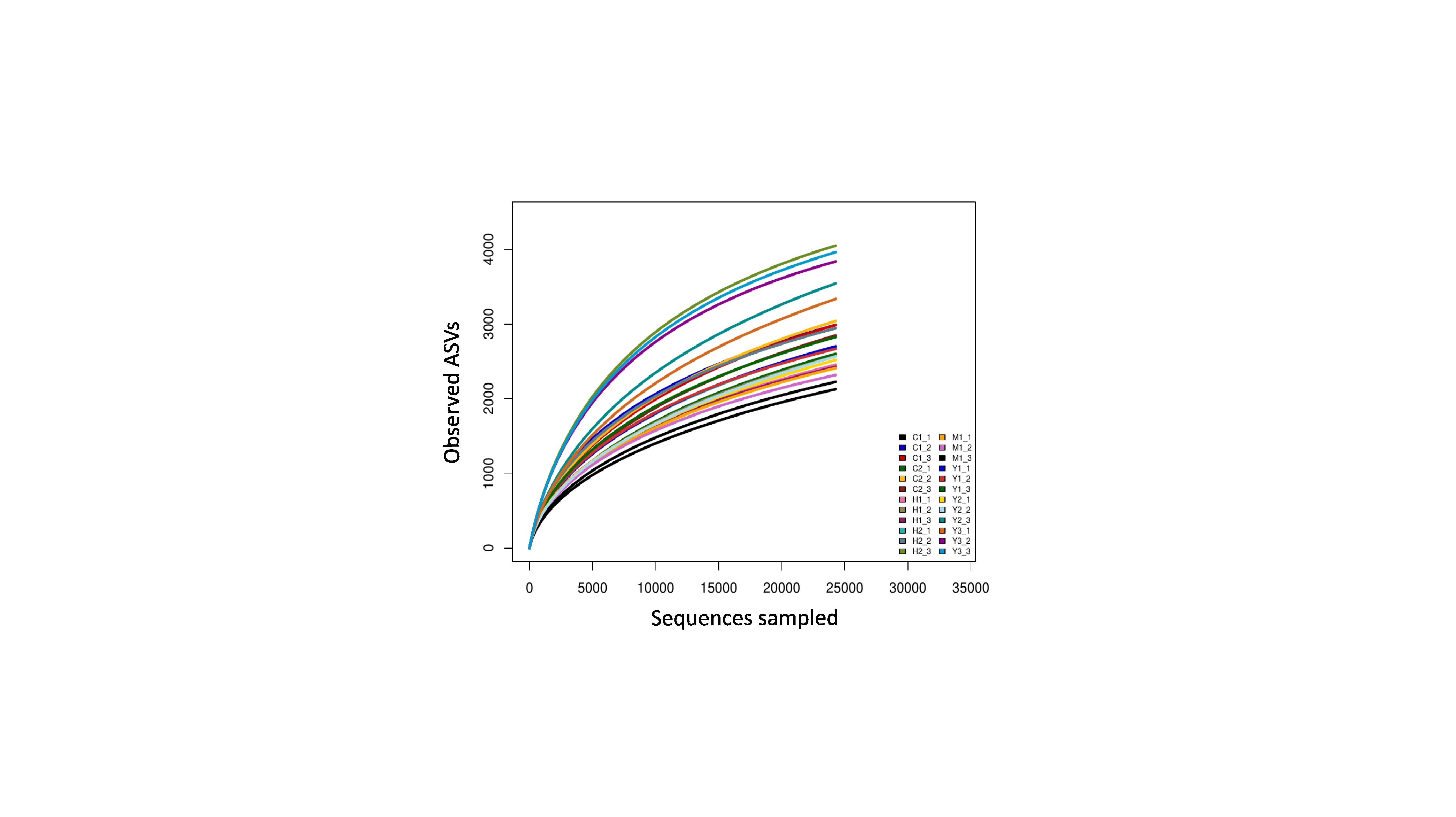

Supplement: Supplementary file 1 [file Image_1.TIFF]
